# The osteopontin-CD44 axis in hepatic cancer stem cells regulates IFN signaling and HCV replication

**DOI:** 10.1038/s41598-018-31421-6

**Published:** 2018-09-03

**Authors:** Takayoshi Shirasaki, Masao Honda, Taro Yamashita, Kouki Nio, Tetsuro Shimakami, Ryougo Shimizu, Saki Nakasyo, Kazuhisa Murai, Natsumi Shirasaki, Hikari Okada, Yoshio Sakai, Tokiharu Sato, Tetsuro Suzuki, Katsuji Yoshioka, Shuichi Kaneko

**Affiliations:** 1 Department of Gastroenterology, Kanazawa University Graduate School of Medical Science, Kanazawa, Japan; 2 Department of Advanced Medical Technology, Kanazawa University Graduate School of Health Medicine, Kanazawa, Japan; 3 Department of General Medicine, Kanazawa University Graduate School of Medical Science, Kanazawa, Japan; 4 Division of Molecular Cell Signaling, Cancer Research Institute, Kanazawa University, Kanazawa, Japan; 5 Department of Virology and Parasitology, Hamamatsu University School of Medicine, Hamamatsu, Japan

## Abstract

Osteopontin (OPN) is involved in cell proliferation, migration, inflammation, and tumor progression in various tissues. OPN induces stemness by interacting with CD44, but the functional relevance of OPN-mediated interferon (IFN) signaling and hepatitis C virus (HCV) replication in stem cell populations remains unclear. In this study, we investigated the effect of OPN on HCV replication and IFN signaling in cancer stem cells (CSCs) positive for epithelial cell adhesion molecule (EpCAM) and CD44. We show that the EpCAM^+^/CD44^+^ CSCs show marked HCV replication when compared to EpCAM^−^/CD44^−^ cells. In addition, OPN significantly enhances this HCV replication in EpCAM^+^/CD44^+^ CSCs and markedly suppresses IFN-stimulated gene expression. The GSK-3β inhibitor BIO increases the EpCAM^+^/CD44^+^ CSC population and OPN expression and impairs IFN signaling via STAT1 degradation. Taken together, our data suggest that OPN enhances HCV replication in the EpCAM^+^/CD44^+^ CSCs, while it also negatively regulates the IFN signaling pathway via inhibition of STAT1 phosphorylation and degradation. Therefore, OPN may represent a novel therapeutic target for treating HCV-related hepatocellular carcinoma.

## Introduction

Hepatitis C virus (HCV) infection, as the major cause of hepatocellular carcinoma (HCC)^1–3^, was estimated to be responsible for 745,000 deaths in 2012^4^. Exclusion of the virus is effective in preventing the hepatic pathogenesis caused by viral infection. Recently, highly efficient and direct-acting antiviral agents (DAAs) have been able to eliminate HCV from infected livers in more than 90% of cases^5,6^. However, an emergence of HCC at a rate of about 1% per year is now reported in HCV-infected livers, even following successful elimination of HCV^7–9^. Therefore, new therapeutic strategies are urgently needed to prevent HCV infection, HCC recurrence, and hepatocarcinogenesis.

Osteopontin (OPN) is a multifunctional cytokine expressed in a variety of tissues. OPN is involved in normal physiological processes, as well as in numerous pathological conditions, including inflammation, angiogenesis, fibrogenesis, and carcinogenesis^10,11^. In liver diseases, OPN plays a critical role in acute liver injury, viral replication, granuloma formation, liver repair, alcoholic steatohepatitis (ASH), non-alcoholic fatty liver disease (NAFLD), fibrosis, and HCC^12–14^. OPN contains an arginine-glycine-aspartic acid (RGD) sequence, which interacts with αvβ1, αvβ3, αvβ5, and α8β1 integrins^15^. It also contains a serine-valine-valine-tyrosine-glycine-leucine-arginine (SVVYGLR) sequence, which interacts with α9β1 and α4β1 integrins^16^. In addition to these interactions with integrins, OPN also reportedly interacts with CD44^17^.

CD44 is a multistructural and multifunctional transmembrane glycoprotein with involvement in lymphocyte activation, hematopoietic differentiation, inflammation, bacterial infection, and cancer^18^. Recent work has identified CD44 as the most common marker for cancer stem cells (CSCs) in several human cancers, including breast^19^, gastric^20^, colon^21^, prostate^22^, colorectal^23^, pancreatic^24^, and head and neck squamous cell carcinomas^25^. CD44 has a pivotal role in regulating the properties of CSCs, including their self-renewal, tumor initiation, metastasis, and chemoradioresistance^26^.

Other recent research has further indicated that HCC conforms to the CSC hypothesis, whereby a small subset of cells with stem cell features drives tumor initiation, metastasis, and chemoradioresistance^27^. In HCC, an enrichment of several stem cell markers, including CD133, CD90, CD13, epithelial cell adhesion molecule (EpCAM), CD44, CD24, and oval cell marker OV6, is reported in certain side populations of CSCs^28,29^. However, CSCs represent only a minor population of the cancer cells^30^ and no evidence yet supports a role for CSCs in supporting HCV replication. Therefore, identifying the underlying mechanism of HCV pathogenesis and its relationship to CSCs is an important research challenge.

In the present study, we evaluated the significance of the OPN-CD44 axis for HCV replication in EpCAM^+^/CD44^+^ CSCs. We demonstrated that EpCAM^+^/CD44^+^ CSCs have the potential to support HCV replication by inducing the CD44 ligand OPN, which inactivates interferon (IFN) signaling. We also investigated the role of OPN in the regulation and maintenance of EpCAM^+^/CD44^+^ CSCs.

## Results

### HCV replication is increased in EpCAM^+^/CD44^+^ CSCs

**Figure 1:**
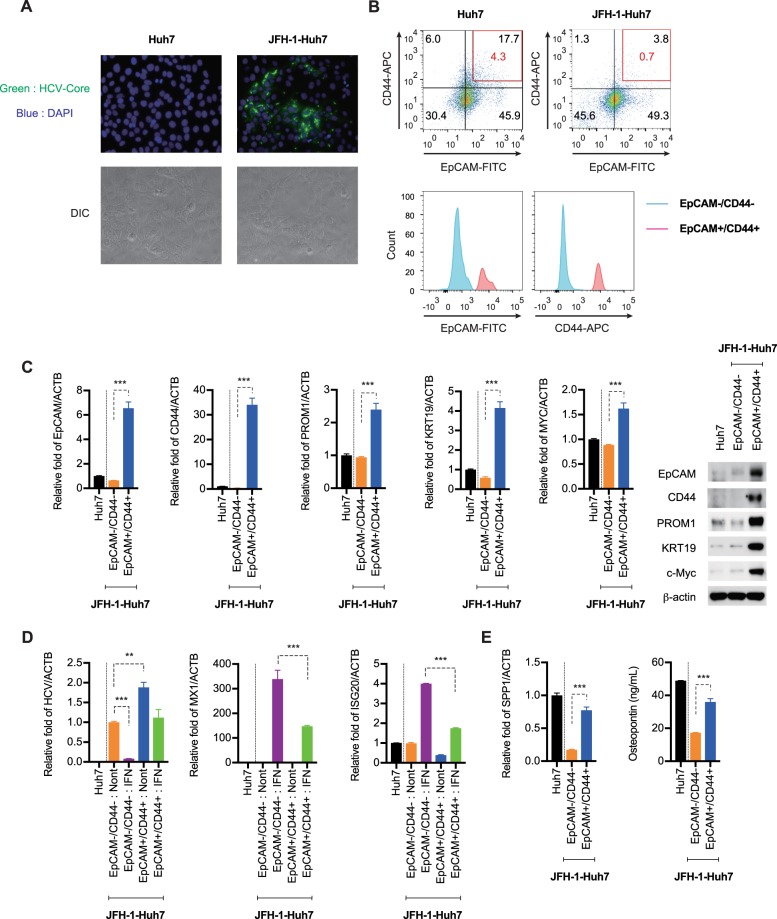
Hepatitis C virus (HCV) replication is increased in EpCAM^+^/CD44^+^ cancer stem cells (CSCs). (A upper panel) Immunofluorescence staining of HCV-core protein in Huh7 cells and JFH-1-Huh7 cells. (A lower panel) DIC image in Huh7 cells and JFH-1-Huh7 cells. (B upper panel) FACS analysis of EpCAM and CD44 expression in Huh7 cells and JFH-1-Huh7 cells. (B lower panel) Histogram of EpCAM^high^/CD44^high^ and EpCAM^−^/CD44^−^ CSC populations after the cell sorting. (C left panel) RTD-PCR analysis of EpCAM, CD44, PROM1, KRT19, and MYC mRNA in EpCAM^+^/CD44^+^ CSCs and EpCAM^−^/CD44^−^ cells. Results were normalized to those of ACTB. (C right panel) EpCAM, CD44, PROM1, KRT19, c-Myc, and β-actin protein levels were determined by immunoblot analysis. The results presented are a representative data and the original full-length blots of the cropped images is provided in Supplemental Fig. S4. (**D**) RTD-PCR analysis of HCV-RNA, MX1, and ISG20 in Huh7, EpCAM^+^/CD44^+^ CSCs, and EpCAM^−^/CD44^−^ cells. Results were normalized to those of ACTB. (**E**) RTD-PCR analysis (left) and ELISA analysis (right) of the level of OPN in Huh7, EpCAM^+^/CD44^+^ CSCs, and EpCAM^−^/CD44^−^ cells. The data are represented as means ± SEM from three independent experiments. **P < 0.01, ***P < 0.001.

We used JFH-1-Huh7 cells^31^, which are Huh7 cells that are continuously infected by the JFH-1 HCV strain. The cells were maintained in normal medium by passaging every week for approximately 6 months. HCV-core protein was detected in JFH-1-Huh7 cells, but not Huh7 cells (Fig. 1A). We first used FACS to evaluate the frequencies of EpCAM^+^/CD44^+^ CSCs in Huh7 cells and JFH-1-Huh7 cells at passage 10. As shown in Fig. 1B, the JFH1-Huh7 cell population consisted of 3.8% EpCAM^+^/CD44^+^ and 45.6% EpCAM^−^/CD44^−^ cells and the Huh7 cell population consisted of 17.7% EpCAM^+^/CD44^+^ and 30.4% EpCAM^−^/CD44^−^ cells. We then used FACS to obtain enriched EpCAM^high^/CD44^high^ (0.7%) and EpCAM^−^/CD44^−^ CSC populations from the JFH1-Huh7 cell cultures (Fig. 1B Histogram), and we evaluated the expression patterns of hepatic stem/maturation markers (EpCAM, CD44, PROM1, KRT19 and MYC) in both populations. These markers were strongly up-regulated in EpCAM^high^/CD44^high^ CSCs when compared with EpCAM^−^/CD44^−^ cells (Fig. 1C). HCV replication was also higher in EpCAM^high^/CD44^high^ CSCs than in EpCAM^−^/CD44^−^ cells and IFN-α significantly increased the level of ISGs in the EpCAM^−^/CD44^−^ CSCs and suppressed HCV replication. Interestingly, the induction of ISGs in EpCAM^high^/CD44^high^ CSCs following IFN treatment was lower than in EpCAM^−^/CD44^−^ cells (Fig. 1D). We also evaluated the OPN mRNA and protein levels by RTD-PCR and ELISA in the two CSC populations and Huh7 cells. As shown in Fig. 1E, the levels of OPN mRNA and protein was higher in EpCAM^high^/CD44^high^ CSCs and Huh7 cells than in EpCAM^−^/CD44^−^ cells.

Some studies have shown an induction of OPN following liver injury, inflammation, and HCV infection^32,33^. We therefore examined whether HCV replication induced OPN expression. Synthetic RNAs of JFH-1 and a translation incompetent JFH-1 (JFH-1-338U)^34^, which has a single-nucleotide mutation from adenine to uracil at the position of nucleotide 338 in the 5′ nontranslated RNA, were transfected into Huh7 cells and the levels of OPN mRNA were evaluated by RTD-PCR. As shown in Supplementary Fig. S1A, expression of OPN was increased in the JFH-1 replicating Huh7 cells (Fig. S1A).

We obtained further confirmation of this increased OPN expression with another strain of HCV, H77S.3/GLuc^35^. The H77S.3/GLuc genome has the Gaussia Luciferase (GLuc) sequence, followed by a foot-and-mouth disease virus 2A autoprotease sequence, inserted in frame between the p7 and NS2 sequences of the H77S.3 strain to allow monitoring of HCV-RNA replication. The synthetic RNAs of H77S.3/GLuc and a replication-incompetent RNA genome, H77S.3/GLuc-AAG, which has a lethal mutation in its RNA polymerase, were transfected into Huh7.5 cells and the levels of OPN protein was evaluated by ELISA. As shown in Supplementary Fig. S1B, OPN concentrations were increased in the culture medium of H77S.3/GLuc replicating Huh7 cells (Fig. S1B).

### OPN significantly increases HCV replication in EpCAM^+^/CD44^+^ CSCs

**Figure 2:**
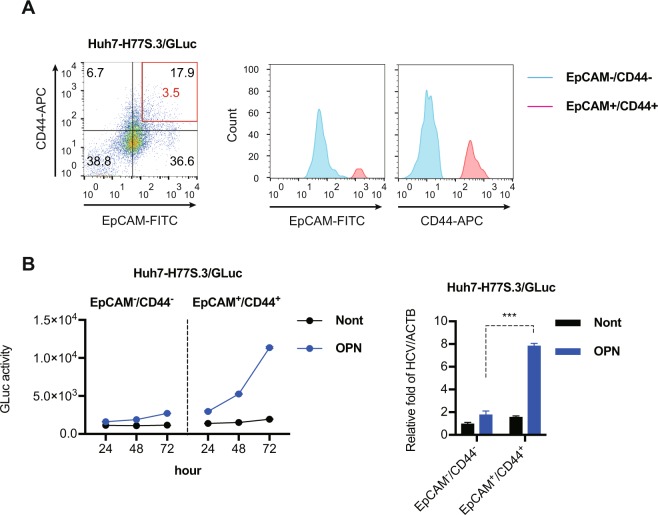
OPN significantly increases HCV replication in EpCAM^+^/CD44^+^ cancer stem cells (CSCs). (A left panel) FACS analysis of EpCAM and CD44 expression in H77S.3/GLuc replicating Huh7 cells 24 h after H77S.3/GLuc-RNA transfection. (A right panel) Histogram of EpCAM^high^/CD44^high^ and EpCAM^−^/CD44^−^ CSC populations after the cell sorting. (**B**) Huh7 cells were transfected with H77S.3/GLuc-RNA, and 24 h later, EpCAM^+^/CD44^+^ cells and EpCAM^−^/CD44^−^ cells were isolated using a BD FACSAria II cell sorting system. At 24 h after sorting, recombinant OPN protein (100 ng/mL) was added (left) The medium was collected and replaced with fresh medium every 24 h until 72 h. GLuc activity was determined at each time point. (right) RTD-PCR analysis of HCV-RNA in EpCAM^+^/CD44^+^ CSCs and EpCAM^−^/CD44^−^ cells 72 h after OPN treatment. Results were normalized to those of ACTB. The data are represented as means ± SEM from three independent experiments. ***P < 0.001.

These initial results indicated that OPN increases HCV replication in EpCAM^+^/CD44^+^ CSCs. We tested this hypothesis by transfecting Huh7 cells with the synthetic RNAs of H77S.3/GLuc and then used FACS to sort enriched EpCAM^high^/CD44^high^ (3.5%) and EpCAM^−^/CD44^−^ populations from the H77S.3/GLuc replicating Huh7 cells (Fig. 2A). At 1 day after sorting, we treated the EpCAM^high^/CD44^high^ CSCs and EpCAM^−^/CD44^−^ cells with recombinant OPN protein and monitored HCV replication with the GLuc assay and RTD-PCR. Recombinant OPN protein significantly increased GLuc activity in EpCAM^high^/CD44^high^ CSCs in a time-dependent manner, but had no effect on expression in EpCAM^−^/CD44^−^ cells (Fig. 2B). OPN also significantly increased the level of HCV-RNA in the EpCAM^high^/CD44^high^ CSCs (Fig. 2B), and this was further confirmed in H77S.3/GLuc replicating Huh7.5 cells (Fig. S2A and B). These results indicated that OPN increases HCV replication in EpCAM^+^/CD44^+^ CSCs through the OPN-CD44 axis.

### BIO augments the EpCAM^+^/CD44^+^ CSC population and increases HCV replication though STAT1 inactivation

**Figure 3:**
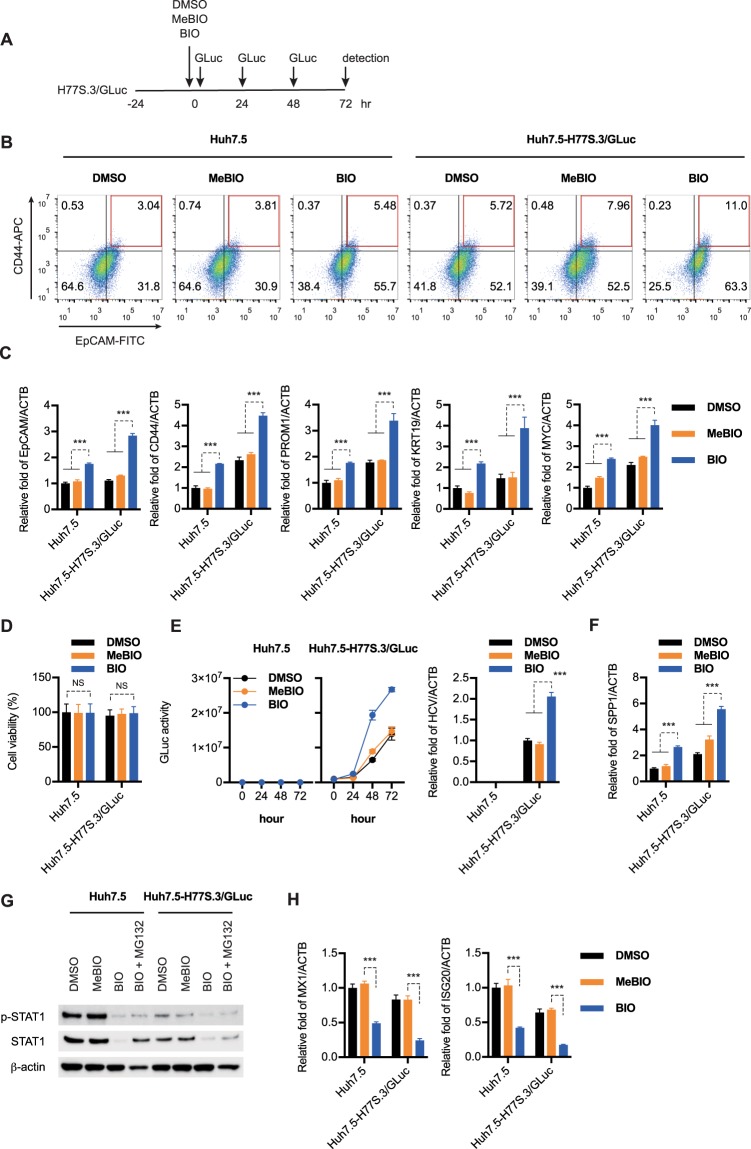
BIO augments the production of EpCAM^+^/CD44^+^ cancer stem cells (CSCs) and increases HCV replication though STAT1 inactivation. (**A**) Schematic of experimental design. (**B**) FACS analysis of EpCAM and CD44 expression in Huh7.5 cells and H77S.3/GLuc replicating Huh7.5 cells 72 h after DMSO, BIO or MeBIO treatment. (**C**) Huh7.5 cells were transfected with H77S.3/GLuc-RNA and 24 h later, DMSO, BIO or MeBIO (2 μmol/L) was added. RTD-PCR was then used to analyze EpCAM, CD44, PROM1, KRT19, and MYC mRNA expression in DMSO, BIO or MeBIO treated cells. Results were normalized to those of ACTB. (**D**) Cytotoxicity was analyzed by MTT assays after inoculation with DMSO, BIO or MeBIO for 72 h. (**E**) (left) The medium was collected and replaced with fresh medium every 24 h until 72 h. GLuc activity was determined at each time point. (right) RTD-PCR analysis of HCV-RNA in DMSO, BIO or MeBIO treated cells, 72 h after treatment. (**F**) RTD-PCR analysis of SPP1 mRNA in DMSO, BIO or MeBIO treated cells. Results were normalized to those of ACTB. (**G**) 72 h after DMSO, BIO (in the presence or absence of MG132) or MeBIO inoculation, phospho-STAT1, STAT1, and β-actin protein levels were determined by immunoblot analysis. The results presented are a representative data and the original full-length blots of the cropped images is provided in Supplemental Fig. S4. (**H**) RTD-PCR analysis of MX1 and ISG20 mRNA in DMSO, BIO or MeBIO treated cells. Results were normalized to those of ACTB. The data represent means ± SEM from three independent experiments. ***P < 0.001.

We examined the role of OPN in EpCAM^+^/CD44^+^ CSCs using the GSK-3β inhibitor BIO, which activates Wnt/β-catenin signaling and maintains embryonic stem cells in an undifferentiated state^36^. We first transfected Huh7.5 cells with the synthetic RNAs of H77S.3/GLuc, and then treated H77S.3/GLuc-replicating Huh7.5 cells with DMSO, with BIO, and with methylated BIO (MeBIO) as a control (Fig. 3A). As shown in Fig. 3B, BIO treatment increased EpCAM^+^/CD44^+^ CSC populations and a greater EpCAM, CD44, PROM1, KRT19, and MYC expression was induced with BIO than with DMSO and MeBIO (Fig. 3C). Evaluation of cell viability with the MTT assay 72 h after DMSO, BIO, and MeBIO treatment revealed no cell viability changes within this period (Fig. 3D). Interestingly, GLuc activity and the levels of HCV-RNA were significantly higher after BIO treatment than after DMSO and MeBIO treatment (Fig. 3E). BIO also induced the expression of OPN (SPP1) mRNA (Fig. 3F).

We explored the mechanism of OPN enhancement of HCV replication in EpCAM^+^/CD44^+^ CSCs by examining the IFN response in these CSCs. The JAK/STAT signaling pathway is essential for anti-HCV activity. IFN-α and IFN-β bind the IFN-α receptor (IFNAR) and activate the receptor-associated protein tyrosine kinases JAK1 and TYK2, which phosphorylate STAT1 and STAT2. Tyrosine-phosphorylated STAT1 and STAT2 dimerize and translocate to the nucleus, where they assemble with IRF9 to form a trimolecular complex that binds to IFN-stimulated response elements (ISREs) and directly activate the transcription of interferon stimulated genes (ISGs)^37^.

Some studies have reported that OPN induces STAT1 degradation through the ubiquitin–proteasome pathway^38,39^. We found that BIO significantly decreased the amounts of activated STAT1 (p-STAT1), as well as the amounts of total STAT1 (Fig. 3G). Interestingly, BIO-dependent degradation of STAT1 in Huh7.5 cells and H77S.3/GLuc-replicating Huh7.5 cells was partially attenuated by proteasome inhibitor MG132 treatment (Fig. 3G). We also found that BIO significantly suppressed the expression of MX1 and ISG20 when compared to the MeBIO and DMSO (Fig. 3H). These results suggest that BIO augments the EpCAM^+^/CD44^+^ CSC population and increases HCV replication though STAT1 degradation.

### BIO augments the EpCAM^+^/CD44^+^ CSC population and attenuates the IFN response

**Figure 4:**
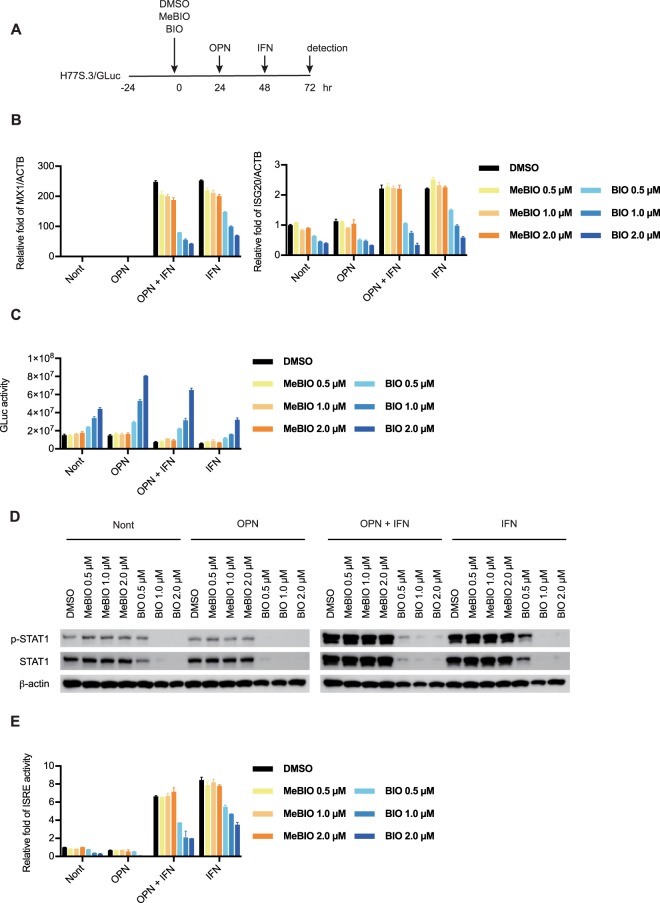
BIO augments the production of EpCAM^+^/CD44^+^ cancer stem cells (CSCs) and attenuates the interferon (IFN) response. (**A**) Schematic of the experimental design. (**B**) Huh7.5 cells were transfected with H77S.3/GLuc-RNA and 24 h later, DMSO, BIO (0.5, 1, 2 μmol/L), or MeBIO (0.5, 1, 2 μmol/L) was added. At 24 h after treatment, recombinant OPN protein (100 ng/mL) was added and at 48 h after treatment, IFN-α (100 IU/mL) was added. RTD-PCR was used to analyze MX1 and ISG20 mRNA expression. Results were normalized to those of ACTB. (**C**) The medium was collected and replaced with fresh medium every 24 h until 72 h. GLuc activity was determined at 72 h. (**D**) Phospho-STAT1, STAT1, and β-actin protein levels were determined by immunoblot analysis 24 h after IFN-α treatment. The results presented are a representative data and the original full-length blots of the cropped images is provided in Supplemental Fig. S4. (**E**) IFN-α stimulation and ISRE reporter activities 24 h after IFN-α treatment.

Next, we examined the effect of BIO on IFN response. We transfected Huh7.5 cells with the synthetic RNAs of H77S.3/GLuc then treated H77S.3/GLuc replicating Huh7.5 cells with DMSO, BIO and the control methylated BIO (MeBIO) in a dose-dependent manner. At 24 h after BIO treatment, we treated with recombinant OPN and then treated cells with IFN-α (Fig. 4A). As shown in Fig. 4B, the induction of ISGs (ISG20 and MX1) by IFN-α treatment was significantly reduced in the presence of BIO in a dose-dependent manner (Fig. 4B). Consistent with these results, GLuc activity was significantly increased in the presence of BIO (Fig. 4C). The induction of pSTAT1, as well as the increased amounts of total STAT1, following IFN-α stimulation was prevented by BIO treatment, but not by MeBIO and DMSO treatments (Fig. 4D). Consistent with these results, IFN-α induced ISRE-dependent transcriptional activity, as measured using an ISRE-luciferase reporter assay, and this induction was significantly suppressed by BIO treatment (Fig. 4E). Interestingly, the effect of BIO was enhanced by OPN treatment (Fig. 4B–E). These results suggest that BIO augments the EpCAM^+^/CD44^+^ CSC population and impairs IFN signaling through inactivation of STAT1 and ISRE activity.

### OPN regulates stemness of EpCAM^+^/CD44^+^ CSCs

**Figure 5:**
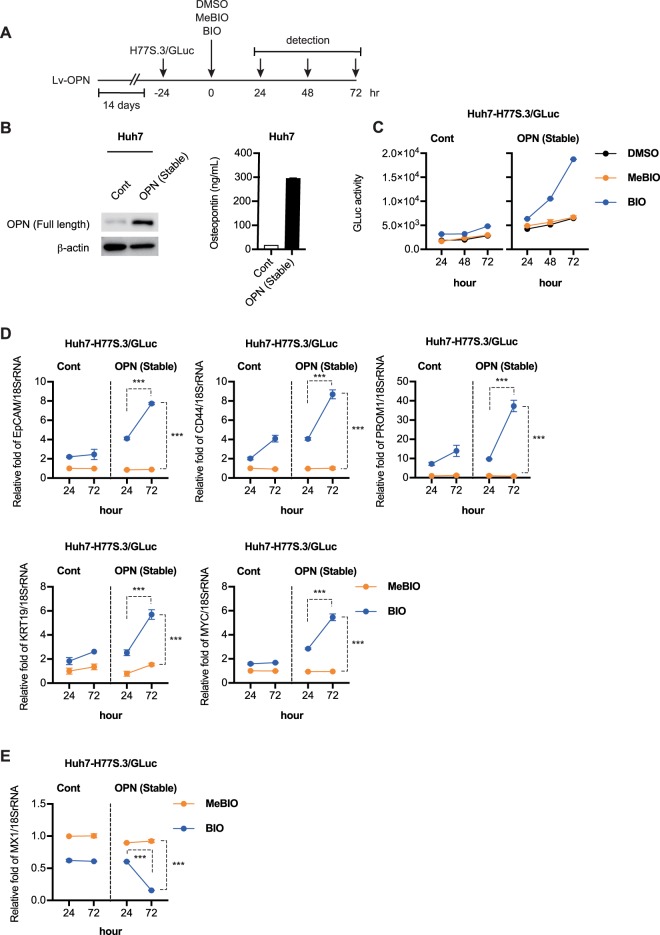
Osteopontin (OPN) regulates stemness of EpCAM^+^/CD44^+^ cancer stem cells (CSCs). (**A**) Schematic of experimental design. (**B**) Immunoblot analysis (left) and ELISA analysis (right) of the level of OPN in Huh7 and OPN overexpressing Huh7 cells. The results presented are a representative data and the original full-length blots of the cropped images is provided in Supplemental Fig. S4. (**C**) Lentivirus-mediated OPN expressing Huh7 cells were transfected with H77S.3/GLuc-RNA and 24 h later, DMSO, BIO or MeBIO (2 μmol/L) was added. The medium was collected and replaced with fresh medium every 24 h until 72 h. GLuc activity was determined at each time point. (**D**) RTD-PCR analysis of EpCAM, CD44, PROM1, KRT19, and MYC mRNA in BIO or MeBIO treated cells at 24 and 72 h. Results were normalized to those of 18SrRNA. (**E**) RTD-PCR analysis of MX1 mRNA in BIO or MeBIO treated cells at 24 and 72 h. Results were normalized to those of 18SrRNA. The data represent means ± SEM from three independent experiments. ***P < 0.001.

We explored the role of OPN in EpCAM^+^/CD44^+^ CSCs by evaluating the effect of its overexpression in Huh7 cells. We used lentiviral vectors to express full length OPN protein in Huh7 cells. Huh7 cells were infected with HEK293T cells produced lentivirus at an moi of 10. As shown in Fig. 5B, we observed significantly increased expression of OPN in Huh7 cells and elevated levels of OPN in the culture medium (Fig. 5B). We then transfected OPN overexpressing Huh7 cells with the synthetic RNAs of H77S.3/GLuc and treated those cells with DMSO, BIO, and MeBIO (Fig. 5A). BIO increased GLuc activity and the level of HCV-RNA when compared to the DMSO and MeBIO treated cells. The OPN overexpressing Huh7 cells showed significantly accelerated GLuc activity following BIO treatment (Fig. 5C). We also observed strong up-regulation of the hepatic stem cell markers (EpCAM, CD44, PROM1, KRT19, and MYC) in the OPN overexpressing Huh7 cells following BIO treatment (Fig. 5D). Consistent with these results, OPN expression strongly suppressed the expression of MX1 in the BIO treated Huh7 cells (Fig. 5E).

Taken together, our data suggest a pivotal role for OPN in regulating the stemness of EpCAM^+^/CD44^+^ CSCs, which leads to inactivation of IFN signaling and enhances HCV replication.

## Discussion

HCV is a hepatotropic virus that causes liver cirrhosis and hepatocellular carcinoma. Therefore, eradication of HCV has been recognized as a reasonable way to prevent disease progression, hepatic complications, and HCC. Direct-acting antivirals (DAA) can cure the majority of patients with chronic hepatitis C (CHC) and hepatocellular carcinoma (HCC)^40^. However, HCC is now emerging in HCV-infected livers at a rate of about 1% per year, even after successful elimination of HCV^9^. Therefore, the development of HCC after undergoing DAA treatment remains a critical issue. In the present study, we focused our attention on CSCs, as HCC is proposed to develop from CSCs, even though they represent a very minor portion of the HCC cell population^28^. However, to our knowledge, HCV replication in CSCs is still poorly understood. Here, we showed the significance of the OPN-CD44 axis for HCV replication in EpCAM^+^/CD44^+^ CSCs.

Some studies have reported elevations of OPN mRNA expression and plasma OPN levels in CHC and HCC patients^41,42^. For example, Iqbal *et al*. reported that HCV-induced AP-1 and Sp1 in the OPN promoter enhanced OPN expression^43^. In the present study, we also showed that HCV infection significantly induced OPN mRNA and protein expression (Fig. S1). Because OPN plays a crucial role in tumor progression and metastasis through binding to CD44^44,45^, we compared the levels of OPN and the capacity for HCV replication in EpCAM^+^/CD44^+^ CSCs and EpCAM^−^/CD44^−^ cells. This study is the first to demonstrate significantly higher OPN mRNA and protein levels and greater HCV replication in EpCAM^+^/CD44^+^ CSCs than in EpCAM^−^/CD44^−^ cells (Fig. 1D and E). These results suggest that OPN levels correlate positively with HCV replication and that OPN is involved in HCV replication and maintenance of CSC features in EpCAM^+^/CD44^+^ CSCs.

CD44 was identified as a CSCs marker in many tumors, including HCC^26^. In HCC, several other markers were also identified, including EpCAM, CD133, CD90, CD24, and CD13. Ma *et al*. reported higher tumorigenicity for CD133^+^ cells than for CD133^−^ cells *in vitro* and *in vivo*^46^. Similarly, Yang *et al*. reported higher tumorigenicity for CD90^+^ than for CD90^−^ cells in a panel of liver cell lines^47^. Interestingly, they also found that most of the CD90^+^ cells also expressed CD44. In 2009, Yamashita *et al*. reported that EpCAM^+^ hepatocellular carcinoma cells showed high tumorigenicity and had stem and progenitor cell features^48^. They clearly showed that EpCAM has a potential to be a direct transcriptional target in the Wnt/β-catenin signaling pathway that participates in governing the self-renewal of cancer cells. They also reported that the transcription factor SALL4 was activated in EpCAM^+^/AFP^+^ HCC and that SALL4 regulated HCC cell stemness by the activation of EpCAM, CK19, and CD44 to impart highly tumorigenic and invasive properties to the cells^49^.

Our results and these reports suggested that the OPN-CD44 axis in EpCAM^+^/CD44^+^ CSCs was important in mediating HCV replication and the expression of CSCs features. Our subsequent experiments confirmed that OPN dramatically increased HCV replication in EpCAM^+^/CD44^+^ CSCs when compared with EpCAM^−^/CD44^−^ cells (Figs 2 and S2). Choi *et al*. previously treated bulk Huh7 and Huh7.5 cells with recombinant OPN protein and monitored HCV replication and found that OPN enhanced HCV replication in the JFH-1-infected cells^41^.

We also confirmed this phenomenon using sub-genomic or full-genomic replicons. MH14C cells are a HCV (genotype 1b) sub-genomic replicon with a modified structure in which the EMCV internal ribosomal entry site (IRES) is replaced with HCV IRES. The JFH-1-Huh7 cells are Huh7 cells that are continuously infected with the JFH-1 (genotype 2a) strain. Huh7-H77S.3/GLuc2A cells and Huh7.5-H77S.3/GLuc2A cells are H77S.3/GLuc replicating cells, where H77S.3/GLuc is a modified full-genomic H77S.3 (genotype 1a) isolate that expresses Gaussia luciferase (GLuc) as a fusion with its polyprotein. When we treated both these cell lines with recombinant OPN protein, we found that OPN enhanced HCV replication (Fig. S3), and the magnitude of the OPN effect on HCV replication in bulk cells was two times greater than in non-treated cells. We demonstrated a five-fold greater HCV replication in OPN treated EpCAM^+^/CD44^+^ CSCs than in OPN treated EpCAM^−^/CD44^−^ cells, supporting the idea that the OPN-CD44 axis was critical for the propagation and pathogenesis of HCV in CSCs.

We then explored potential mechanisms for the enhanced HCV replication capacity of the EpCAM^+^/CD44^+^ CSCs using BIO treatment. Sato *et al*. previously reported that activation of the canonical Wnt pathway maintained the self-renewal of human and mouse embryonic stem cells and that Wnt pathway activation by BIO maintained the undifferentiated phenotype in these cells^36^. Similarly, Yamashita *et al*. reported that BIO increased the EpCAM^+^ cell population in HuH1 and HuH7 cells when compared with the control cells treated with methylated BIO (MeBIO)^48^. In the present study, we found that BIO induced the expression of the cancer stem cell markers EpCAM, CD44, KRT19, and MYC above the levels seen in MeBIO controls without inducing cytotoxicity (Fig. 3B). Importantly, BIO promoted a time-dependent increase in HCV replication above that observed in the MeBIO controls (Fig. 3D). BIO also induced the expression of OPN (SPP1) mRNA (Fig. 3E), providing convincing support that CSCs have a positive effect on HCV replication.

BIO treatment also significantly decreased the amounts of activated STAT1 (p-STAT1), as well as amounts of total STAT1 (Fig. 3F). Consistent with these results, BIO suppressed the expression of ISG20 above that observed in the MeBIO controls (Fig. 3G). The IFNs, comprising type I (IFN-α/β), type II (IFN-γ), and type III (IFN-λ), are critical cytokines of the antiviral response and the host immune response^50^. Upon IFN receptor activation, downstream Janus kinase (JAK) and tyrosine kinase (TYK2) are activated and phosphorylate STAT1 and STAT2 to form activated p-STAT1 and p-STAT2. The activated forms heterodimerize, associate with IFN-regulatory factor 9 (IRF9), and translocate to the nucleus. This complex, called the interferon stimulated gene factor 3 (ISGF3), binds to the promoters of the ISGs. Therefore, STAT1 would appear to be essential for antiviral activity against HCV. Notably, Gao *et al*. reported that OPN induced the ubiquitin-dependent degradation of STAT1 in endotoxin (LPS) stimulated macrophages^38^. That finding strongly supports our results indicating that the OPN-CD44 axis increases HCV replication in EpCAM^+^/CD44^+^ CSCs through STAT1 degradation and suppression of ISG expression.

We also demonstrated that BIO treatment impaired the IFN response by decreasing the amount of STAT1 (Fig. 4). Type I IFNs (e.g., IFN-α/β) have several biological functions, including antiviral responses, immune responses, and anti-proliferative activity^51^. This IFN has therefore been widely used for the treatment of viral infections and cancer. In HCC, several studies have reported that the IFN-based combination chemotherapy improved the overall survival of patients with HCC when compared with IFN-α monotherapy^52,53^. IFN-based treatment also decreased the HCC incidence in patients with HCV-related cirrhosis, and the HCC recurrence rate in patients with HCV-related HCC following curative HCC therapy^54^. Recently, Hou *et al*. showed significant down-regulation of the expression of an IFN-stimulated gene, retinoic acid-inducible gene-I (RIG-I), in human HCC tissues. RIG-I is a critical sensor for host recognition of HCV, and patients with low RIG-I expression had a shorter survival and poorer response to IFN-α therapy^55^. Hou *et al*. also demonstrated that RIG-I enhanced the IFN-α response by strengthening STAT1 activation. We therefore suggest that the OPN-CD44 axis in CSCs might be a negative regulator for IFN-based combination chemotherapy in HCC.

We also examined the effect of OPN on CSC diversification. We found that OPN significantly accelerated the BIO-induced CSCs phenotypes (Fig. 5C). Consistent with this result, OPN overexpressing Huh7 cells significantly increased HCV replication when ISG induction was suppressed by BIO treatment. Several studies have reported that OPN-CD44 signaling contributes to the maintenance of cancer stem cell phenotypes and promotes aggressive tumor growth^56,57^, in agreement with our findings.

Collectively, the results of our study highlight a new role for OPN in supporting HCV replication in EpCAM^+^/CD44^+^ CSCs through a reduction in STAT1 activation. We also provide evidence that OPN has the potential to maintain CSC phonotypes, and we identify the OPN-CD44 pathway as a potential target for regulating HCV replication and stemness in HCC cells.

## Methods

### Cells

Huh7 cells, JFH-1-Huh7 cells, Huh7.5 cells, and MH14C cells were maintained in DMEM (Thermo Fisher Scientific) supplemented with 10% fetal bovine serum (Thermo Fisher Scientific), 1% L-glutamine (Thermo Fisher Scientific), and 1% penicillin/streptomycin (Thermo Fisher Scientific) in a humidified atmosphere of 5% CO_2_ at 37 °C.

### Reagents

Recombinant Human OPN protein was purchased from R&D Systems (Minneapolis, MN). Recombinant IFN-α, MG132, BIO, and MeBIO were purchased from Sigma-Aldrich (St. Louis, MO).

### HCV replication assay

The HCV replication assay was performed by transfecting Huh7.5 or Huh7 cells with JFH-1, JFH-1-338U, H77S.3/GLuc, or H77S.3/GLuc-AAG RNA. For RNA transfection, the cells were washed with phosphate-buffered saline (PBS) and resuspended in complete growth medium. The cells were then pelleted by centrifugation (1,400 g for 4 min at 4 °C), washed twice with ice-cold PBS, and resuspended in ice-cold PBS at a concentration of 7.5 × 10^6^ cells/0.4 mL. The cells were mixed with 10 μg of the RNA transcripts, placed into 2-mm-gap electroporation cuvettes (BTX Genetronics, San Diego, CA), and electroporated with five pulses of 99 μs at 750 V over 1.1 s in an ECM 830 device (BTX Genetronics). Following a 10-min recovery period, the cells were mixed with complete growth medium and plated.

### Cell sorting

Cells were trypsinized, washed, and resuspended in Hank’s Balanced Salt Solution supplemented with 1% HEPES and 2% FBS. Cells were then incubated on ice for 30 min with an FITC-conjugated anti-EpCAM antibody (Clone Ber-EP4; DAKO, Carpinteria, CA) and APC-conjugated anti-CD44 antibody (eBioscience, San Diego, CA). The resulting EpCAM^+^/CD44^+^ cells and EpCAM^−^/CD44^−^ cells were isolated using a BD FACSAria II cell sorting system (BD Biosciences, San Jose, CA).

### Cytotoxicity assay

H77S.3/GLuc RNA transfected Huh7.5 cells were seeded in 12-well plates at a density of 5 × 10^5^ cells per well and treated with BIO and MeBIO. After 3 days of treatment, the cell viability was determined by using a Cell Counting Kit-8 (DOJINDO, Kumamoto, Japan) according to the manufacturer’s instructions.

### Viral vector preparation

Human Full length OPN were amplified with PCR and DNA fragment was inserted into pCL20c vector at the AgeI and NotI sites. We used pCL20c-GFP vector as a control. The pCL20c series of viral vectors was produced by the cotransfection of HEK293T cells with a mixture of four plasmids, pCAGkGP1R, pCAG4RTR2, pCAG-VSV-G, and pCL20c^58^. The medium containing the viral particles was harvested 64 h after transfection. The medium samples were filtered through 0.45-μm membranes, centrifuged at 25,000 rpm for 90 min, resuspended in PBS, and stored as frozen aliquots at −80 °C until use.

### Quantitative RTD-PCR

Total RNA was isolated using the GenEluteTM Mammalian Total RNA Miniprep Kit (Sigma-Aldrich Japan K.K., Tokyo, Japan), and cDNA was synthesized using the High Capacity cDNA reverse transcription kit (Applied Biosystems, Carlsbad, CA). RTD-PCR was performed using a 7500 Real Time PCR System (Applied Biosystems, Carlsbad, CA) according to the manufacturer’s instructions. The primer pairs and probes for EpCAM, CD44, PROM1, KRT19, MYC, SPP1, ISG20, MX1, ACTB, and 18SrRNA were obtained from the TaqMan assay reagents library. HCV-RNA was quantified by RTD-PCR analysis using the primer set 5′-CGGGAGAGCCATAGTGG-3′ and 5′-AGTACCACAAGGCCTTTCG-3′ and probe 5′-CTGCGGAACCGGTGAGTACAC-3′ to sequences located in the 5′ UTR.

### Western blotting and immunofluorescence staining

Western blotting was performed as previously described^59^. Cells were washed in phosphate buffered saline (PBS) and lysed in radioimmunoprecipitation assay (RIPA) buffer containing complete Protease Inhibitor Cocktail and PhosSTOP (Roche Applied Science, Indianapolis, IN). Membranes were blocked in Blocking One solution (Nacalai Tesque, Kyoto, Japan) and the expression of EpCAM, CD44, PROM1, KRT19, c-Myc, OPN, phospho-STAT1, STAT1, and β-actin was evaluated with a rabbit anti-EpCAM antibody (Abcam, Cambridge, MA), a rabbit anti-OPN antibody (IBL, Gunma, Japan), mouse anti-CD44 antibody, rabbit anti-PROM1 antibody, rabbit anti-KRT19 antibody, rabbit anti-c-Myc antibody, rabbit anti-phospho-STAT1 antibody, rabbit anti-STAT1 antibody and rabbit anti-β-actin antibody (Cell Signaling Technology Inc., Danvers, MA), respectively. For immunofluorescence staining, the cells were washed twice with PBS and fixed in 4% paraformaldehyde for 15 min at room temperature. After washing again with PBS, the cells were permeabilized with 0.05% Triton X-100 in PBS for 15 min at room temperature. They were then incubated in a blocking solution (10% FBS and 5% BSA in PBS) for 30 min and with the anti-HCV-core monoclonal antibodies (Abcam, Cambridge, MA). The fluorescent secondary antibodies were Alexa 488-conjugated anti-mouse IgG antibodies (Invitrogen, Carlsbad, CA). Imaging was performed with a BIOREVO fluorescence microscope (Keyence Corporation, Osaka, Japan).

### *Gaussia* luciferase assay

Cell culture supernatant fluids were collected at intervals after RNA transfection and fresh medium was added to the cells. Secreted GLuc was measured as described previously.

### ELISA

OPN in the culture medium was measured using the commercially available OPN ELISA kit (IBL, Gunma, Japan) in accordance with the manufacturer’s instructions.

### ISRE reporter assay

Construction of the interferon stimulated response element (ISRE)-Luc reporter plasmid was described previously^31^. Huh7.5 cells were transfected with the ISRE-Luc reporter plasmid 24 hours before BIO and MeBIO treatment, and then cells were treated with IFN-α. After 24 hours, luciferase activities were measured using the Dual Luciferase assay system (Promega, Madison, WI). Values were normalized to the luciferase activity of the co-transfected pGL4.75 Renilla luciferase-expressing plasmid (Promega).

### Statistics

The results are expressed as the mean ± standard deviation. Significance was defined as p < 0.05 and was confirmed by Student’s t test or a paired t test. Statistical analyses were performed using GraphPad Prism 7 (La Jolla, CA, USA).

## Electronic supplementary material

Supplementary Information
