# Supplementary material for: The osteopontin-CD44 axis in hepatic cancer stem cells regulates IFN signaling and HCV replication
Source: Sci Rep. 2018 Sep 3;8:13143. doi: 10.1038/s41598-018-31421-6 (PMC6120883; doi:10.1038/s41598-018-31421-6)
Supplement: Supplementary file 1 — Supplementary Information [file 41598_2018_31421_MOESM1_ESM.docx]

**Supplementary Information**

**Osteopontin-CD44 axis in the hepatic cancer stem cells regulates IFN signaling and HCV replication**

Takayoshi Shirasaki^1,2^, Masao Honda^1,2^, Taro Yamashita^1,3^, Kouki Nio^1^, Tetsuro Shimakami^1^, Ryougo Shimizu^1,2^, Saki Nakasyo^1,2^, Kazuhisa Murai^1,2^, Natsumi Shirasaki^1^, Hikari Okada^1^, Yoshio Sakai^1^, Tokiharu Sato^4^, Tetsuro Suzuki^5^, Katsuji Yoshioka^4^ & Shuichi Kaneko^1^

^1^Department of Gastroenterology, Kanazawa University Graduate School of Medical Science, Kanazawa, Japan.

^2^Department of Advanced Medical Technology, Kanazawa University Graduate School of Health Medicine, Kanazawa, Japan.

^3^Department of General Medicine, Kanazawa University Graduate School of Medical Science, Kanazawa, Japan.

^4^Division of Molecular Cell Signaling, Cancer Research Institute, Kanazawa University, Kanazawa, Japan.

^5^Department of Virology and Parasitology, Hamamatsu University School of Medicine, Hamamatsu, Japan.

**Supplementary Figure S1**

**Figure S1. OPN is induced in HCV infection.** (A) The synthetic RNAs of JFH-1 and a translation incompetent JFH-1 (JFH-1-338U) were transfected into Huh7 cells. The RNA levels of HCV, SPP1, and β-actin were measured by RTD-PCR, with the mRNA levels of HCV and SPP1 normalized to those of β-actin. (B) The synthetic RNAs of H77S.3/GLuc and a replication-incompetent RNA genome (H77S.3/GLuc-AAG) were transfected into Huh7.5 cells. (left) The medium was collected and replaced with fresh medium every 24 h until 72 h. GLuc activity was determined at 72 h. (right) OPN in the culture medium was measured by ELISA. The data are represented as means ± SEM from three independent experiments. ***P<0.001.

**Supplementary Figure S2**

**Figure S2. OPN significantly increases HCV replication in EpCAM^+^/CD44^+^ CSCs.** (A) FACS analysis of EpCAM and CD44 expression in H77S.3/GLuc replicating Huh7.5 cells 24 h after H77S.3/GLuc-RNA transfection. (B) Huh7.5 cells were transfected with H77S.3/GLuc-RNA and 24 h later, EpCAM^+^/CD44^+^ cells and EpCAM^-^/CD44^-^ cells were isolated using a BD FACSAria II cell sorting system. 24h after sorting, recombinant OPN protein was added at the concentrations of 100 ng/ml. (left) The medium was collected and replaced with fresh medium every 24 h until 72 h. GLuc activity was determined at each time point. (right) RTD-PCR analysis of HCV-RNA in EpCAM^+^/CD44^+^ CSCs and EpCAM^-^/CD44^-^ cells 72 h after OPN treatment. Results were normalized to those of ACTB. The data are represented as means ± SEM from three independent experiments. **P<0.01.

**Supplementary Figure S3**

**Figure S3. OPN slightly enhances HCV replication in bulk HCV replicating cells.** (A) MH14C cells were treated with recombinant OPN protein and 48 h after treatment, the RNA levels of HCV and β-actin were measured by RTD-PCR, with the mRNA levels of HCV and normalized to those of β-actin. (B) JFH-1-Huh7 cells were treated with recombinant OPN protein and 48 h after treatment, the RNA levels of HCV and β-actin were measured by RTD-PCR, with the mRNA levels of HCV and normalized to those of β-actin. (C) Huh7 cells were transfected with H77S.3/GLuc-RNA and 24 h later, recombinant OPN protein was added at the concentrations of 100 ng/ml. The medium was collected and replaced with fresh medium every 24 h until 48 h. GLuc activity was determined at 48 h. (D) Huh7.5 cells were transfected with H77S.3/GLuc-RNA and 24 h later, recombinant OPN protein was added at the concentrations of 100 ng/ml. The medium was collected and replaced with fresh medium every 24 h until 48 h. GLuc activity was determined at 48 h. The data are represented as means ± SEM from three independent experiments. **P<0.01, ***P<0.001.

**Supplementary Figure S4**

**Full unedited gel for Figure 1C**

The red marks indicate the bands reported in Figure 1C.

**Full unedited gel for Figure 3G**

The red marks indicate the bands reported in Figure 3G.

**Full unedited gel for Figure 4D**

The red marks indicate the bands reported in Figure 4D.

**Full unedited gel for Figure 5B**

The red marks indicate the bands reported in Figure 5B.
